# Safety and efficacy of different surgical approaches in single-port robot-assisted radical prostatectomy based on propensity score matching analysis

**DOI:** 10.1007/s00345-026-06583-y

**Published:** 2026-07-02

**Authors:** Jin-Chun Qi, Liang Liu, Sheng Tang, Hao-Xuan Yang, Lorenzo Santodirocco, Ying-Ran Xia, Luca Alfredo Morgantini, Ruben Sauer Calvo, Chang-Bao Qu, Ya-Xuan Wang, Simone Crivellaro

**Affiliations:** 1 Department of Urology, the Second Hospital of Hebei Medical University, No.215 Heping West Road, Shijiazhuang, Hebei China; 2 Department of Urology, University of Illinois at Chicago, Chicago, IL USA; 3 Urology Department, Chongqing University Central Hospital, Fourth People’s Hospital of Chongqing, Chongqing Emergency Medical Center, Chongqing University, Chongqing, China; 4 Department of Maternal-Infant and Urological Sciences, Sapienza University of Rome, Umberto I Hospital, Rome, Italy; 5 Department of Urology, Shijiazhuang People’s Hospital, Shijiazhuang, 050011 Hebei China

**Keywords:** Single-port robotic surgery, Radical prostatectomy, Extraperitoneal approach, Transperitoneal approach, Surgical outcomes, Urinary continence

## Abstract

**Purpose:**

The optimal surgical approach (transperitoneal vs. extraperitoneal) for single-port robot-assisted radical prostatectomy (SP-RARP) remains a subject of ongoing debate, with limited high-quality evidence to compare their safety and efficacy. This study aimed to compare the safety, effectiveness, and functional outcomes of transperitoneal (SP-TPRP) and extraperitoneal (SP-EPRP) approaches in SP-RARP using propensity score matching (PSM).

**Methods:**

This retrospective study analyzed 211 consecutive patients who underwent SP-RARP at a tertiary referral center (2018–2024): 58 SP-TPRP and 153 SP-EPRP. PSM was performed to balance baseline covariates. Perioperative outcomes were compared between the two groups. Time to urinary continence recovery was assessed using Kaplan-Meier analysis.

**Results:**

After PSM, 55 patients per group were analyzed. The SP-EPRP group demonstrated significantly higher estimated blood loss (150.0 vs. 100.0 mL, *p* = 0.021) and a shorter hospital stay (1 vs. 2 days, *p* < 0.001), and lower pain scores(0.0 vs. 4.0, *p* < 0.001). Gastrointestinal complications occurred exclusively in SP-TPRP (*n* = 5), while lymphoceles only in SP-EPRP (*n* = 4). No significant differences were found in operative time, overall complications, short-term oncological outcomes or function recovery. Kaplan-Meier analysis showed no significant difference in time to continence recovery between approaches (*p* = 0.32).

**Conclusions:**

Both approaches achieve comparable oncological outcomes, perioperative safety, and functional recovery. SP-EPRP was associated with shorter hospital stay and lower early postoperative pain, but higher estimated blood loss. Both approaches appear clinically feasible, and prospective studies with longer follow-up are warranted to validate these findings.

## Introduction

Prostate cancer is a leading malignancy among men worldwide in both incidence and mortality [1]. For localized disease, radical prostatectomy remains a cornerstone curative treatment [2]. The technique has continuously evolved toward minimally invasive approaches: from open surgery to conventional laparoscopy, and subsequently to robot-assisted radical prostatectomy (RARP). Compared with traditional approaches, RARP provides equivalent oncological control and functional preservation while offering advantages including reduced blood loss, less postoperative pain, shorter hospital stays, and quicker recovery [3, 4].

The advent of the purpose-designed single-port (SP) robotic platform gave rise to single-port robot-assisted radical prostatectomy (SP-RARP), with the first clinical experience using the da Vinci SP surgical system reported by Kaouk et al. after FDA clearance [5]. This technique utilizes a single small incision for all instruments, theoretically minimizing abdominal wall trauma and offering potential benefits in cosmetic outcomes, postoperative pain, and recovery [6, 7].

Regarding the choice of surgical approach, transperitoneal and extraperitoneal routes are the two most established pathways in RARP. Systematic reviews and meta-analyses have indicated that while oncological efficacy and long-term functional outcomes are largely comparable between the two approaches, the extraperitoneal pathway is often associated with shorter operation times and a reduced risk of intra-abdominal complications such as ileus, potentially facilitating a smoother recovery after surgery [8]. In SP-RARP, several comparative studies have examined extraperitoneal and transperitoneal access and have provided important evidence suggesting that the extraperitoneal approach may offer selected recovery-related advantages, including shorter hospital stay and reduced analgesic requirements [9–11]. Nevertheless, additional single-center data from a cohort characterized by a relatively homogeneous surgical platform, consistent institutional perioperative workflow, serially assessed functional outcomes, and granular characterization of complication types may further clarify approach-specific perioperative and recovery profiles.

Therefore, this study employed a propensity score matching (PSM) analysis on data from a cohort of 211 patients. Data were analyzed to compare the transperitoneal and extraperitoneal approaches in SP-RARP regarding perioperative safety and efficacy outcomes, with additional assessment of postoperative recovery, urinary continence over time and approach-specific complication patterns.

## Patients and methods

### Study design and population

Data from a prospectively maintained, IRB-approved database were retrospectively analyzed, including all patients who underwent SP-RARP between December 2018 and December 2024. All procedures were performed by three high-volume robotic surgeons at a tertiary referral center (University of Illinois Medical Center, Chicago, IL, USA) using previously described techniques [9]. The study was conducted in accordance with the ethical standards of the institutional and national research committee (IRB: 2017 − 0152) and the 1964 Helsinki Declaration. Written informed consent was obtained from all participants.

Inclusion criteria were: (1) age ≥ 18 years, (2) SP-RARP as the sole procedure, and (3) absence of severe cardiopulmonary disease contraindicating surgery. Exclusion criteria included: (1) concurrent major abdominal surgery, (2) severe cardiopulmonary disease precluding safe surgery, and (3) incomplete clinical data. Of 225 initially screened patients, 211 were included for final analysis. Patients were categorized into transperitoneal (SP-TPRP, *n* = 58) and extraperitoneal (SP-EPRP, *n* = 153) groups.

### Data collection

Patient data were collected via EPIC System^®^ and reviewed. Baseline characteristics included age, BMI, prostate volume, ASA grade, age-adjusted Charlson Comorbidity Index (CCI), Gleason score, and AJCC TNM classification. Intraoperative parameters included operative time, estimated blood loss (EBL), pelvic lymph node dissection (PLND), lymph node yield, intraoperative complications, nerve-sparing status, and blood transfusion. PLND was performed at the operating surgeon’s discretion based on clinical risk assessment and intraoperative judgment. Postoperative complications were recorded by type and severity using the Clavien-Dindo classification. All surgical specimens were examined by dedicated uropathologists to determine histological type, pathological T and N stages, extraprostatic extension, surgical margin condition (all positive margins were non-focal [≥ 3 mm]), margin location, and the International Society of Urological Pathology (ISUP) group. Follow-up data included hospital stay, serum prostate-specific antigen (PSA) at approximately 40 days post-surgery, immediate and postoperative urinary continence (6 weeks, 3 months, 6 months), erectile function at 3 months, and pain scores assessed using the Numeric Rating Scale (NRS) both in the immediate postoperative period and at discharge. PSA persistence was defined as PSA ≥ 0.2 ng/mL at the approximately 40-day postoperative assessment. Urinary continence recovery was defined as complete pad-free status within 24 h, in accordance with the 2012 Pasadena Consensus Panel criteria [12]. Immediate postoperative continence was defined as the absence of urinary leakage after catheter removal and was recorded based on the first post-catheter-removal continence assessment available in the database. Erectile function recovery was defined as the ability to achieve and maintain an erection sufficient for satisfactory sexual intercourse, irrespective of phosphodiesterase type 5 inhibitor use.

Postoperative PSA, urinary continence, and erectile function data were incomplete for some patients because of the retrospective follow-up design. Descriptive summaries of these outcomes were based on the evaluable patients for each endpoint and time point. Missing postoperative outcome data were not imputed, and the number of evaluable patients was reported for each postoperative endpoint.

### Statistical analysis

Statistical analyses were performed using R software (version 4.5.1). PSM was employed to minimize confounding using 1:1 nearest-neighbor matching without replacement, with a caliper width of 0.2 times the standard deviation of the logit of the propensity score. Matching covariates included age, BMI, ASA grade, age-adjusted CCI, prostate volume, Gleason score, and clinical TNM stage. Continuous variables are presented as medians with interquartile ranges (IQR), and categorical variables are presented as frequencies and percentages. In the unmatched cohort, continuous variables were compared using the Wilcoxon rank-sum test, and categorical variables were compared using the chi-square test or Fisher’s exact test, as appropriate. For comparisons in the matched cohort, the paired structure generated by 1:1 PSM was accounted for in the statistical analysis. Paired continuous variables were compared using the Wilcoxon signed-rank test. Paired binary categorical variables were compared using the exact McNemar test, and paired multicategory variables were compared using the Stuart-Maxwell test. For paired comparisons of postoperative PSA, urinary continence, and erectile function outcomes, only matched pairs in which both patients had available data for the corresponding endpoint and time point were included in the paired test. Lymph node yield was analyzed only among patients who underwent PLND and was compared using the Wilcoxon rank-sum test. Event-level complication types were summarized descriptively and compared using Fisher’s exact test. Covariate balance before and after matching was assessed using standardized mean differences (SMDs). A Love plot was used to graphically summarize covariate balance before and after PSM. For continence recovery, time-to-event analysis was performed using the Kaplan-Meier method. Time to continence recovery was defined as the interval from surgery to the first documented postoperative assessment at which the continence criterion was met. Patients without documented continence recovery were censored at their last available continence assessment. Curves were compared using the log-rank test. A two-sided *p* < 0.05 was considered statistically significant.

## Results

### Baseline characteristics of participants

**Fig. 1:**
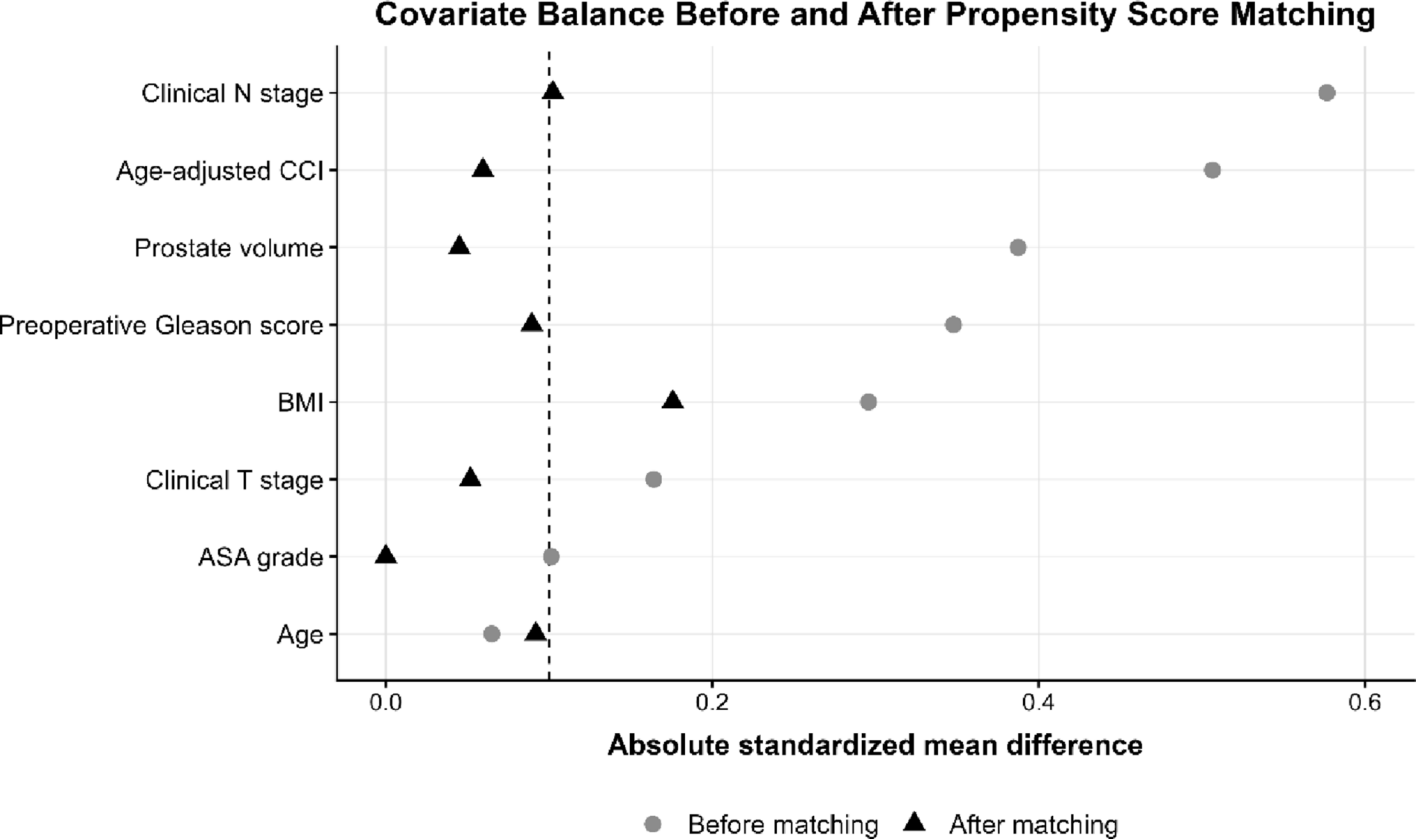
Love Plot Showing Covariate Balance Before and After Propensity Score Matching

A total of 225 patients who underwent SP-RARP between December 2018 and December 2024 were initially identified. After considering the inclusion and exclusion criteria and omitting patients lacking essential data, 211 patients were deemed eligible for PSM. Following 1:1 PSM, 110 patients were included in the final matched group, comprising 55 patients in SP-TPRP group and 55 patients in SP-EPRP group. Baseline characteristics before and after PSM are shown in Table 1. In the unmatched cohort, several baseline covariates were imbalanced between groups, most notably age-adjusted CCI, clinical N stage, prostate volume, BMI, and preoperative Gleason score. Covariate balance improved substantially following PSM, with all post-matching SMDs below 0.20 and no statistically significant differences observed between groups for baseline characteristics (all *P* > 0.05). This improvement was further demonstrated by the Love plot (Fig. 1).

**Table 1:** Baseline characteristics before and after propensity score matching according to surgical approach

Variable	Before PSM				After PSM			
	Control group - Transperitoneal Approach (*N* = 58)	Observation group - Extraperitoneal Approach (*N* = 153)	*p*-value	SMD	Control group - Transperitoneal Approach (*N* = 55)	Observation group- Extraperitoneal Approach (*N* = 55)	*p* value	SMD
Age, Median (IQR) (years)	63.5 (59.0, 70.0)	64.0 (60.0, 68.0)	0.882	0.065	63.0 (58.0, 70.0)	64.0 (60.0, 70.0)	0.652	0.092
BMI, Median (IQR) (kg/m^2^)	29.7 (25.8, 32.9)	27.9 (24.6, 32.6)	0.125	0.296	29.4 (25.7, 32.9)	29.4 (25.8, 32.8)	0.792	0.176
ASA grade			0.534	0.101			1.000	0.000
<= ASA II	26 (44.8%)	61 (39.9%)			24 (43.6%)	24 (43.6%)		
>= ASA III	32 (55.2%)	92 (60.1%)			31 (56.4%)	31 (56.4%)		
Age-adjusted CCI			0.002	0.507			0.843	0.059
0–3	2 (3.4%)	16 (10.5%)			2 (3.6%)	1 (1.8%)		
4–5	11 (19.0%)	57 (37.3%)			10 (18.2%)	10 (18.2%)		
>=6	45 (77.6%)	80 (52.3%)			43 (78.2%)	44 (80.0%)		
Prostate size, (mL) Median (IQR)	49.1 (41.2, 76.0)	48.0 (38.0, 61.0)	0.090	0.387	49.0 (40.0, 73.4)	49.0 (38.0, 69.0)	0.666	0.045
Clinical T stage			0.479	0.164			0.948	0.052
cT1	43 (74.1%)	110 (71.9%)			41 (74.5%)	40 (72.7%)		
cT2	10 (17.2%)	21 (13.7%)			9 (16.4%)	9 (16.4%)		
cT3	5 (8.6%)	22 (14.4%)			5 (9.1%)	6 (10.9%)		
Clinical N stage			< 0.001	0.576			1.000	0.102
cN0	57 (98.3%)	111 (72.5%)			54 (98.2%)	53 (96.4%)		
cN1	1 (1.7%)	5 (3.3%)			1 (1.8%)	2 (3.6%)		
cNx	0 (0.0%)	37 (24.2%)			0 (0.0%)	0 (0.0%)		
Preoperative Gleason score			0.056	0.348			0.935	0.089
6	10 (17.2%)	23 (15.0%)			10 (18.2%)	9 (16.4%)		
7 (3 + 4)	33 (56.9%)	61 (39.9%)			30 (54.5%)	30 (54.5%)		
7 (4 + 3)	5 (8.6%)	32 (20.9%)			5 (9.1%)	7 (12.7%)		
>=8	10 (17.2%)	37 (24.2%)			10 (18.2%)	9 (16.4%)		

*BMI*: Body mass index; *IQR*: Interquartile range; *ASA*: American Society of Anesthesiologists; *CCI*: Charlson comorbidity index

### Perioperative outcomes

The perioperative results are detailed in Table 2. Operative time was similar between the SP-TPRP and SP-EPRP groups (308.0 vs. 314.0 min, *p* = 0.738), whereas EBL was higher in the SP-EPRP group (150.0 vs. 100.0 mL, *p* = 0.021). The PLND rate was higher in the SP-EPRP group, though not statistically significant (56.4% vs. 41.8%, *p* = 0.169). Among patients who underwent PLND, median lymph node yield was numerically lower in the SP-EPRP group but did not differ significantly between groups (8.0 vs. 12.0, *p* = 0.142). Intraoperative complications, nerve-sparing status, blood transfusion, and postoperative complication severity were comparable between groups. The distribution of complication types differed between groups (*p* = 0.038), with gastrointestinal complications occurring only in the SP-TPRP group (5 cases), and lymphoceles occurring only in the SP-EPRP group (4 cases). Other complications were sparsely and similarly distributed between the two groups.

**Table 2:** Intraoperative outcomes and postoperative complications according to surgical approach in the matched cohort

Characteristics	Control group - Transperitoneal Approach (*n* = 55)			Observation group - Extraperitoneal Approach (*n* = 55)		*p* value			
Operative time, (minutes) Median (IQR)	308.0 (278.0, 356.0)			314.0 (278.0, 353.0)		0.738			
Estimated blood loss, (mL) Median (IQR)	100.0 (50.0, 200.0)			150.0 (100.0, 300.0)		0.021			
Lymphadenectomy, n (%)						0.169			
No	32 (58.2%)			24 (43.6%)					
Yes	23 (41.8%)			31 (56.4%)					
Median Lymph node yield, *n* = 54, n | Median (IQR), (n)	23 | 12.0 (9.0, 14.0)			31 | 8.0 (5.0, 13.0)		0.142			
Intraoperative complications, n (%)						0.508			
No	49 (89.1%)			52 (94.5%)					
Yes	6 (10.9%)			3 (5.5%)					
Nerve sparing procedure, n (%)						0.578			
No	21 (38.2%)			16 (29.1%)					
Unilateral	3 (5.5%)			3 (5.5%)					
Bilateral	31 (56.4%)			36 (65.5%)					
Intraoperative blood transfusions, n (%)						1.000			
No	54 (98.2%)			55 (100.0%)					
Yes	1 (1.8%)			0 (0.0%)					
Postoperative complication, n (%)						0.406			
No	31 (56.4%)			36 (65.5%)					
Clavien-Dindo < III	18 (32.7%)			12 (21.8%)					
Clavien-Dindo ≥ III	6 (10.9%)			7 (12.7%)					
Complication type						0.038			
Total of complications	27			22					
Urine leak	2			3					
Gastrointestinal complications	5			0					
Renal complications	2			0					
Urinary tract infection	5			5					
Lymphocele	0			4					
Other	13			10					

### Pathological outcomes and postoperative recovery

**Fig. 2:**
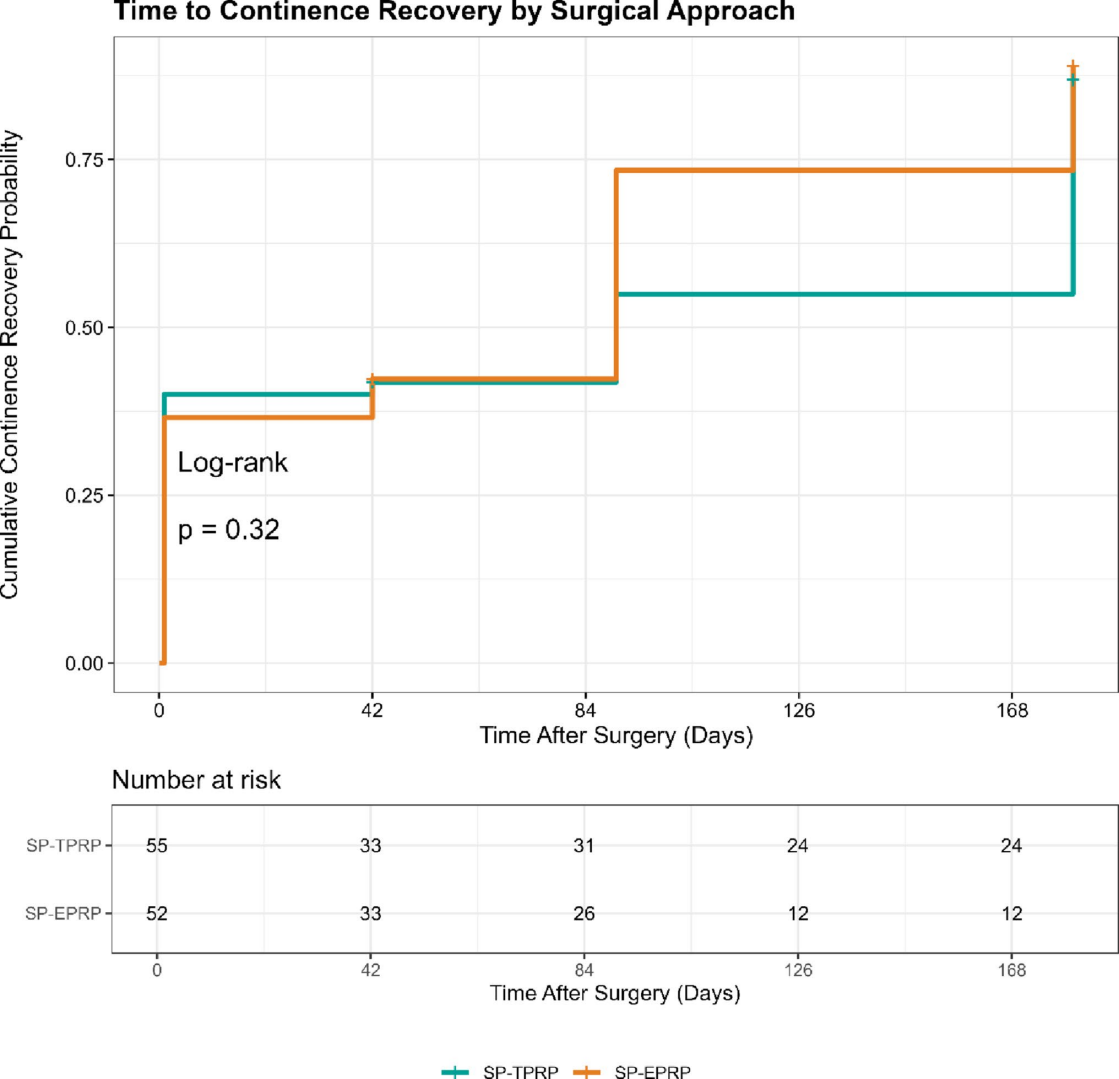
Kaplan-Meier Estimates of Urinary Continence Recovery After Single-Port Robot-Assisted Radical Prostatectomy

Pathological examination results and postoperative recovery metrics are presented in Table 3. Histological type, pathological T and N stages, extraprostatic extension, surgical margin status and laterality, ISUP grade group, 40-day PSA level, and PSA persistence were comparable between groups. At approximately 40 days post-surgery, PSA measurements were available for 100 patients, including 52 in the SP-TPRP group and 48 in the SP-EPRP group. Median PSA levels did not differ significantly between groups (0.0 vs. 0.0 ng/mL, *p* = 0.702). Likewise, the rate of PSA persistence was similar between the SP-TPRP and SP-EPRP groups (8/52 [15.4%] vs. 10/48 [20.8%], *p* = 0.774). Regarding postoperative recovery, the SP-EPRP group had significantly shorter hospital stay (1 vs. 2 days, *p* = 0.002) and lower immediate postoperative pain scores (0.0 vs. 5.0, *p* = 0.040), whereas pain scores at discharge were similar. Three-month erectile function recovery was evaluable in 84 patients and did not differ significantly between groups (5/46 [10.9%] vs. 6/38 [15.8%], *p* = 1.000). The urinary continence gradually improved in both groups postoperatively. In the available-case analysis, continence was numerically higher in the SP-EPRP group than in the SP-TPRP group at 3 months, although the difference was not statistically significant (28/53 [52.8%] vs. 30/42 [71.4%], *p* = 0.210). By 6 months, continence rates were comparable between the SP-TPRP and SP-EPRP groups (46/53 [86.8%] vs. 35/41 [85.4%], *p* = 0.727). To account for differences in follow-up availability and censoring, time to continence recovery was further evaluated using Kaplan-Meier analysis, which showed no significant difference between groups (*p* = 0.32; Fig. 2).

## Discussion

This study evaluated the safety and effectiveness of transperitoneal versus extraperitoneal approaches in SP-RARP. Both approaches showed comparable oncological and functional outcomes. Operative time was similar between groups, whereas EBL was higher in the SP-EPRP group. Additionally, the SP-EPRP group demonstrated more favorable early postoperative recovery, as reflected by a shorter hospital stay and lower early postoperative pain scores. The SP-TPRP group experienced more gastrointestinal complications, whereas lymphoceles were only found in the SP-EPRP group.

Operative time did not differ significantly between the SP-EPRP and SP-TPRP groups. EBL was higher in the SP-EPRP group than in the SP-TPRP group. Although the extraperitoneal approach avoids intraperitoneal access and may reduce bowel manipulation, this did not translate into a measurable operative time advantage in the present cohort. However, a recent meta-analysis focusing on SP-RARP found no significant differences in operative time but reported higher intraoperative blood loss with SP-EPRP [11]. Similarly, in the multi-institutional study by Abou Zeinab et al., EBL was higher in the EPRP group than in the TPRP group (150 [100–200] vs. 75 [50–150] mL) [10]. The PLND rate was higher in the SP-EPRP group, though this difference was not statistically significant. The higher EBL observed in the SP-EPRP group may be partly related to the numerically higher proportion of patients undergoing PLND in this group, as prior RARP data have shown that more extensive PLND may increase blood loss while improving lymph node yield [13]. Nevertheless, EBL should be interpreted cautiously because it is influenced by multiple factors, including the extent of nodal dissection, prior abdominal or pelvic surgery, local adhesions, and patient anatomy, rather than the surgical access route alone [14]. Among patients who underwent PLND, the median lymph node yield was numerically lower in the SP-EPRP group but did not differ significantly between groups. This variation may result from the surgeon’s intraoperative assessment of the technical feasibility and oncological value of performing lymph node dissection with each approach. The limited working space in the extraperitoneal approach might lead surgeons to prefer a more focused, limited dissection over an extensive template dissection [15]. Our previous study showed that while the SP-EPRP approach resulted in a lower lymph node yield during PLND, it achieved comparable outcomes to the standard multiport transperitoneal approach in identifying lymph node-positive patients and the number of positive lymph nodes retrieved [16].

Despite the use of different surgical access routes, both approaches achieved comparable short-term oncological control. No significant differences were found in positive surgical margin rates, extraprostatic extension, PSA levels at 40 days postoperatively, or PSA persistence. This aligns with Uy et al.‘s systematic review on multiport RARP, indicating that surgical approach does not compromise tumor resection thoroughness [8]. This shows SP-RARP can meet standard oncological radicality requirements regardless of approach when performed by experienced surgeons. Nevertheless, the available follow-up remains short, and longer-term endpoints such as biochemical recurrence-free survival will be needed to more fully evaluate oncological equivalence between approaches.

**Table 3:** Pathological outcomes and postoperative recovery according to surgical approach in the matched cohort

Characteristics	Control group - Transperitoneal Approach (n = 55)	Observation group - Extraperitoneal Approach (n = 55)	*p* value
Histological type, n (%)			0.625
Acinar adenocarcinoma	54 (98.2%)	52 (94.5%)	
Others	1 (1.8%)	3 (5.5%)	
Pathological stage (T), n (%)			0.369
pT0	1 (1.8%)	1 (1.8%)	
pT2	34 (61.8%)	34 (61.8%)	
pT3	17 (30.9%)	20 (36.4%)	
pT4	3 (5.5%)	0 (0.0%)	
Pathological stage (N), n (%)			0.885
pN0	13 (23.6%)	15 (27.3%)	
pN1	8 (14.5%)	7 (12.7%)	
pNx	34 (61.8%)	33 (60.0%)	
Extraprostatic extension, n (%)			1.000
No	38 (69.1%)	37 (67.3%)	
Yes	17 (30.9%)	18 (32.7%)	
Surgical margin, n (%)			0.423
Negative	40 (72.7%)	41 (74.5%)	
Positive, right	7 (12.7%)	4 (7.3%)	
Positive, left	1 (1.8%)	4 (7.3%)	
Positive, bilateral	7 (12.7%)	6 (10.9%)	
Pathological grade group (ISUP), n (%)			0.981
ISUP 1	10 (18.2%)	9 (18.4%)	
ISUP 2	31 (56.4%)	28 (57.1%)	
ISUP 3	4 (7.3%)	5 (10.2%)	
ISUP 4	6 (10.9%)	5 (10.2%)	
ISUP 5	4 (7.3%)	2 (4.1%)	
PSA at 40 days post-surgery, n = 100, n | Median (IQR) (ng/ml)	52 | 0.0 (0.0, 0.0)	48 | 0.0 (0.0, 0.1)	0.702
PSA persistence, n = 100, n (%)			0.774
No	44 (84.6%)	38 (79.2%)	
Yes	8 (15.4%)	10 (20.8%)	
Length of hospital stay, Median (IQR)(days)	2 (1, 2)	1 (1, 1)	0.002
Pain score at postoperative, Median (IQR)	5.0 (0.0, 7.0)	0.0 (0.0, 5.0)	0.040
Pain score at discharge, Median (IQR)	0.0 (0.0, 3.0)	0.0 (0.0, 3.0)	0.347
Potency in 3 months, n = 84, n (%)			1.000
Yes	5 (10.9%)	6 (15.8%)	
No	41 (89.1%)	32 (84.2%)	
Postoperative continence, n = 100, n (%)			1.000
Yes	22 (41.5%)	19 (40.4%)	
No	31 (58.5%)	28 (59.6%)	
Continence in 6 weeks, n = 107, n (%)			1.000
Yes	23 (41.8%)	22 (42.3%)	
No	32 (58.2%)	30 (57.7%)	
Continence in 3 months, n = 95, n (%)			0.210
Yes	28 (52.8%)	30 (71.4%)	
No	25 (47.2%)	12 (28.6%)	
Continence in 6 months, n = 94, n (%)			0.727
Yes	46 (86.8%)	35 (85.4%)	
No	7 (13.2%)	6 (14.6%)	

*ISUP* International Society of Urological Pathology; *PSA* Prostate specific antigen

One major discovery is that the extraperitoneal approach was associated with favorable early postoperative recovery indicators. Patients in the SP-EPRP group had a median hospital stay one day shorter than the transperitoneal group (1 vs. 2 days) and reported lower postoperative pain scores. These results are consistent with our earlier study showing that the extraperitoneal approach confers shorter hospital stays and less postoperative pain in RARP [9]. Postoperative gastrointestinal dysfunction is a primary non-surgical factor contributing to delayed discharge. The extraperitoneal approach, confined to the extraperitoneal space, avoids entering the abdominal cavity, minimizing bowel manipulation, peritoneal irritation, and postoperative intra-abdominal adhesions. The transperitoneal approach requires pneumoperitoneum establishment and a Trendelenburg position, which can increase intra-abdominal pressure, compromise mesenteric blood flow, and stimulate the vagus nerve - established contributors to postoperative ileus, nausea/vomiting, and delayed pain resolution [17]. Gastrointestinal complications, including ileus (*n* = 2) and nausea/vomiting (*n* = 3), occurred only in the SP-TPRP group. No such complications were reported in the SP-EPRP group. This suggests the extraperitoneal approach may benefit patients with abdominal surgery history or those at high risk for extensive adhesions, especially regarding postoperative gastrointestinal recovery [18]. Although overall complications and major complications did not differ, the complication profiles showed distinct characteristics between groups. Beyond gastrointestinal complications, four lymphocele cases occurred in the SP-EPRP group, with none in the SP-TPRP group. This phenomenon is frequently reported in multiport approach comparisons [19]. The mechanism involves lymphatic fluid absorption by the peritoneal surface in the transperitoneal approach, whereas in the confined extraperitoneal space, lymphatic fluid accumulates and forms cysts [20]. When using the extraperitoneal approach, attention should focus on lymphatic vessel ligation, and preventive measures like intraoperative peritoneal fenestration or adequate postoperative drainage should be considered [21].

Regarding functional outcomes, erectile function recovery at 3 months was limited in both groups (5/46 [10.9%] vs. 6/38 [15.8%]), with no statistically significant difference between approaches. These findings should be interpreted cautiously because erectile function recovery data were affected by incomplete follow-up, and the assessment interval was short. Postoperative erectile function recovery is influenced by multiple factors, including baseline erectile function, nerve-sparing techniques, and surgical approach impact on pelvic neurovascular bundles [22]. Longer follow-up with more complete functional assessment is therefore needed. Regarding urinary continence, both groups demonstrated gradual improvement over time. At 3 months, the SP-EPRP group showed a higher observed continence rate than the SP-TPRP group; however, this difference did not reach statistical significance. At 6 months postoperatively, continence rates were comparable between groups, with most patients achieving satisfactory urinary continence. Kaplan-Meier analysis further showed no significant difference in time to continence recovery between approaches. This finding is consistent with a recent systematic review and meta-analysis of RARP approaches, which reported comparable 6-month continence recovery between extraperitoneal and transperitoneal approaches [23]. Furthermore, Liao et al. reported that their modified maximum peri-prostatic anatomy-preserving robotic-assisted laparoscopic radical prostatectomy (MPAP-RALP) technique facilitated early continence recovery without compromising surgical margin rates, achieving continence rates of 95.1% at 3 months postoperatively [24].

This study has several limitations. First, the retrospective design, even with the application of PSM, cannot eliminate the risk of selection bias and residual confounding. In particular, prior abdominal or pelvic surgery was not consistently available in the database and therefore could not be included as a matching covariate, although it may influence surgical approach selection. Second, as a single-center study conducted by high-volume surgeons, the generalizability of our findings to institutions with varying surgical expertise may be limited. Because the original SP-EPRP cohort was larger than the SP-TPRP cohort, 1:1 PSM excluded a substantial number of SP-EPRP patients. Therefore, the generalizability of the matched-cohort findings to the broader SP-RARP population may be limited. Third, PLND was performed at the operating surgeon’s discretion rather than according to a standardized protocol and was not included as a matching variable. Therefore, nodal outcomes, including pN stage and lymph node yield, should be interpreted cautiously because PLND selection and extent differed between groups. Fourth, postoperative PSA and functional outcomes were affected by incomplete follow-up data. Erectile function recovery was assessed only at the early 3-month time point and was defined clinically rather than using a validated questionnaire, which limits interpretation of potency recovery. The limited follow-up duration also restricts assessment of durable oncological and functional outcomes, including biochemical recurrence, sustained urinary continence, and erectile function recovery. Finally, because no a priori power calculation was performed and no adjustment for multiple comparisons was applied, these findings should be interpreted cautiously and validated in larger prospective studies.

## Conclusion

This study compared transperitoneal and extraperitoneal SP-RARP using PSM. The two approaches yielded comparable oncological outcomes, perioperative safety, and functional recovery. Operative time was similar between groups, whereas SP-EPRP was associated with shorter hospital stay and lower early postoperative pain, but higher EBL. Both approaches appear clinically feasible, and future prospective studies with long-term follow-up are warranted to confirm these findings.

## Data Availability

Supporting data for the findings of this study are accessible upon reasonable request to the corresponding author.
